# The impact of population aging and rural food markets on the dietary quality of rural residents: a micro case study from China

**DOI:** 10.3389/fnut.2026.1757603

**Published:** 2026-06-12

**Authors:** Zichen Wang, Haitao Zhang, Jiahao Zhang, Shan Lu, Wenling Lai, Rui Wang, Jieling Shi, Danqi Wei

**Affiliations:** 1 School of Innovation and Entrepreneurship, Wuchang University of Technology, Wuhan, China; 2 School of Finance and Economics, Jimei University, Xiamen, China

**Keywords:** dietary decision-making, dietary quality, nutrition information, population aging, rural families, rural food market

## Abstract

Against the backdrop of the accelerating aging of the rural population, strengthening the construction of rural food circulation networks holds significant practical significance for improving the dietary quality of rural residents and narrowing the nutritional gap between urban and rural areas. From a food policy and economics perspective, this study investigates how market-based food environments moderate the nutritional consequences of demographic aging. Based on the food consumption survey data of 1,080 rural households in Shandong, Jiangxi and Gansu provinces in 2024, this paper empirically analyzes the influence mechanism of population aging (PA) and rural food markets (RFM) on the dietary quality of rural residents (DQRR) by used the linear regression model. The main research findings are as follows: (1) PA has a significant negative impact on the DQRR, while RFM can effectively promote the improvement of dietary quality for rural residents. This conclusion still holds true after a series of robustness tests. (2) RFM has a positive moderating effect on the negative impact of PA. (3) Food environmental perception, including supply sufficiency, spatial accessibility and economic affordability, is an important mechanism by which PA negatively affects DQRR. (4) Obtaining dietary nutrition information is an important mechanism for the food market to improve the quality of meals. (5) The impact of PA and RFM on the DQRR varies due to the gender of family dietary decision-makers, the strength of family clan networks, and the size of village populations. The new findings of this study help to understand the key influencing factors of the dietary quality of rural residents and provide a basis for food policy design aimed at responding to population aging and promoting the integrated development of urban and rural food markets.

## 1 Introduction

Improving the dietary quality of rural residents is an important part of promoting the comprehensive revitalization of rural areas and the construction of a healthy China. It has long-term and practical significance for improving the family welfare of rural residents and cultivating rural health human capital (1–3). At present, the global society is simultaneously undergoing two profound structural changes: (1) The accelerating trend of population aging (PA) (4); (2) The disease burden caused by changes in dietary patterns is increasing day by day (5). The intersection and interaction of these two major trends not only pose a severe test to the public health systems and social security systems of all countries, but also present a core challenge to the global efforts to achieve the United Nations 2030 Agenda for Sustainable Development and its seventeen Sustainable Development Goals (SDGs). PA, as a major achievement in human social development, is closely related to the health and well-being of SDG 3. This goal explicitly requires ensuring a healthy life for people of all ages. However, due to the decline of physiological functions, the high incidence of chronic diseases and the constraints of social and economic factors, the elderly group has become a high-risk group for malnutrition (6). A low-quality dietary structure, specifically manifested as deficiencies of micronutrients, insufficient intake of dietary fiber, and excessive intake of salt, sugar and unhealthy fats, significantly increases the risk of cardiovascular diseases, diabetes, osteoporosis and cognitive dysfunction in the elderly. This not only directly undermines the quality of life and functional autonomy of the elderly, but is also closely linked to the goal of “eliminating all forms of malnutrition” set by SDG 2. The concept of “hunger” here has transcended the traditional sense of energy deficiency and expanded to include “hidden hunger” such as micronutrient deficiencies and non-communicable diseases related to diet. This article aims to explore the impact of population aging on the dietary quality of rural residents (DQRR) and how to alleviate this negative influence. This study approaches this question from a food policy and economics lens, focusing on the role of rural food markets as an economic infrastructure that can buffer the dietary risks associated with aging.

In 2025, the No. 1 Document issued by the Chinese government sets improving the quality of diet as a policy orientation. It put forward the concepts of ‘vigorously promoting healthy diets' and ‘promoting diets that reduce oil, salt, sugar and increase whole grains”. These policy signals highlight the significant position of optimizing the dietary structure of residents and ensuring their nutritional health in building a healthy China. While nutritional health represents the ultimate outcome, the mechanisms we examine, namely food market availability, household income constraints, and transaction costs, are core themes in food economics and policy research. As a fundamental indicator for measuring the living standards and health status of residents, dietary quality is not only directly related to the balance of individual nutritional intake and the effectiveness of disease prevention (7, 8), but also profoundly affects the level of rural human capital and its accumulation efficiency through intergenerational transmission. At present, there are still prominent problems in the dietary structure of rural residents in China, such as low food diversity, insufficient intake of animal protein, and deficiency of micronutrients (9). Especially under the combined effect of various factors such as the accelerated aging of the population and the imbalance of public services and infrastructure between urban and rural areas, this structural imbalance has gradually evolved into a key bottleneck restricting the improvement of family welfare for rural residents. Against the backdrop of an accelerating aging population, how to improve the dietary quality of rural residents is a key issue that needs to be addressed.

The discussions in the existing literature on the influencing factors of dietary quality of rural residents are mostly limited to micro characteristics such as income level, dietary knowledge, household production, and accessibility to specific markets (10–14). There are also literatures that analyze the correlation between poverty and dietary health (15). However, they generally overlook the changes in demand brought about by the transformation of population structure and the overall supply constraints caused by the incomplete food market, making it difficult to reveal the profound impact of hard constraints such as limited food access channels in an aging society on the dietary quality of rural residents. The incompleteness of the development of food markets in rural areas is a key obstacle restricting the improvement of dietary quality for rural residents (16). However, the limitations of the existing research perspectives often lead policy design to remain at the micro-intervention level, failing to effectively respond to the complex practical demands brought about by the superimposed impact of population aging and underdeveloped markets.

Population aging is reshaping the socio-economic ecology of rural China, and its impact on the dietary quality of residents shows obvious group heterogeneity (17). With a large number of young and middle-aged labor forces migrating to urban and non-agricultural sectors, the aging rate of rural society is accelerating, and the trends of aging and empty-nest are evolving rapidly (18). Data from the China Population and Employment Statistical Yearbook shows that the proportion of the population aged 60 (65) and above in rural areas of China has increased from 17.09% (11.16%) in 2013 to 25.02% (19.31%) in 2022, and the proportion of the elderly aged 80 and above has been continuously rising. The increase in the proportion of elderly people living alone can easily lead to problems such as the solidification of traditional dietary patterns and the narrowing of channels for obtaining dietary knowledge, thereby exacerbating the decline in the family's ability to prepare meals and adjust dietary quality. Specifically, due to the decline in physiological functions, the elderly have a significantly increased demand for nutrients such as protein and vitamins. However, their actual intake levels are constrained by multiple factors such as low market accessibility, weak economic affordability, and information asymmetry, leading to a simultaneous increase in nutritional gaps and the risk of chronic diseases (19).

In the process of building a unified urban and rural market, the food market in towns and townships, by linking the urban and rural food circulation network, is expected to become a key breakthrough to solve the problem of dietary quality for rural residents in the context of population aging (20). China's Ministry of Commerce pointed out that it is necessary to “improve the consumption environment in rural areas” and “meet the upgrading demands of farmers' consumption”, injecting institutional impetus into the functional upgrading of rural markets. Theoretically, rural food markets may bridge the weak links in the urban and rural food supply chain by optimizing supply adequacy, enhancing spatial accessibility, and increasing economic affordability, thereby meeting the local residents' consumption demands for high-nutrition foods. Meanwhile, the dietary nutrition information dissemination network derived from the market agglomeration effect may, through channels such as the demonstration effect of merchants, the peer effect of consumers, and the embedding of public services, potentially improve the dietary cognition and choice preferences of the elderly group, buffer the decline effect of dietary quality in aging families, and provide decision-making references for responding to health challenges brought about by changes in population structure.

To position our study within the existing discourse of food policy and economics, we note that related fields has examined dietary quality through individual level factors such as income, price, nutrition knowledge, and agricultural production diversity (5, 21, 22) . In the era of digital economy, published literature analyzes the positive effect of technological progress on dietary nutrition from the perspective of digital technology, Internet and mobile phone APP, and reveals that technology availability is an important factor for improving residents' health (23–25). There is also a part of the literature that analyzes the external variables that affect food availability and food security from the perspective of the United Nations Sustainable Development Goal 2, and considers population aging as a factor that cannot be ignored (26). We also observed other drivers of food security and dietary nutrition, such as environmental regulation (27), diversity in agricultural production (28), and nutrition education (29). However, the interplay between population aging as a demographic structural change and rural food market development as an economic infrastructure has received limited attention. Pan and Min (30) provided initial evidence, but the mediating pathways of supply sufficiency, spatial accessibility, and economic affordability remain untested together.

Our study contributes to this literature by theoretically and empirically demonstrating that rural food markets can buffer the negative dietary consequences of aging. This mechanism operates through price effects, convenience effects, and information spillover effects, which are distinct from purely behavioral or clinical pathways. The marginal contributions of this article are as follows: (1) This study deepens the understanding of the factors influencing the dietary quality of rural residents. Different from the previous studies' focus on factors such as household income and agricultural production (10, 11, 14). This paper supplements the key influencing factors of the dietary quality of rural residents from the perspectives of population structure change and food market development, which is conducive to further understanding the constraints and driving factors of the dietary quality of rural residents. (2) We have revealed the supplementary role of township food markets in the dietary quality of rural residents. The existing literature mainly focuses on the village-level market, including the influence of rural e-commerce and traditional food markets on the food consumption behavior of rural residents (30, 31). Few studies have explored the possible role of township food markets in improving the dietary quality of rural residents based on the reality that rural food markets are underdeveloped. (3) We analyzed the impact of population aging and the food market in towns and townships on the dietary quality of rural residents and its mechanism. Existing studies mainly explore the direct impact of population structure and external markets on food consumption of rural residents (12, 32). This article further deconstructs the influence mechanism of population aging and rural food markets on the dietary quality of rural residents from two aspects: food environment perception and dietary nutrition information acquisition. The central research question of this paper is as follows: how does population aging affect the dietary quality of rural residents, and can the development of rural food markets buffer this negative impact? To answer this question, we proceed in four steps. (1) We estimate the baseline effects of population aging and rural food markets on dietary quality. (2) We test whether rural food markets positively moderate the negative impact of aging. (3) We examine two mediating pathways: food environment perception and dietary nutrition information acquisition. (4) We explore heterogeneity by gender of dietary decision makers, clan network strength, and village population size. This structure follows the standard empirical approach in food policy and economics research.

To ensure a clear understanding of our study design, we provide additional justification for the selection of Shandong, Jiangxi, and Gansu provinces. These three provinces were chosen to represent the diverse environmental and resource constraints that characterize rural China. Shandong Province, located in the eastern coastal region, features abundant water resources, flat terrain, and a highly developed agricultural economy. It represents resource rich rural areas with relatively strong market access. Jiangxi Province, situated in the central and southern region, has moderate resource endowments, hilly terrain, and a mixed agricultural structure. It captures intermediate conditions between resource rich and resource scarce areas. Gansu Province, located in the northwestern arid and semi arid region, suffers from severe water scarcity, fragile ecological conditions, and lower agricultural productivity. It exemplifies resource constrained rural areas where food market development faces significant challenges. Importantly, Jiangxi and Shandong or our country's important grain production base. In these two provinces, the food supply is very sufficient. By covering these three distinct provincial contexts, our sampling framework captures a wide spectrum of environmental and economic conditions, thereby enhancing the external validity of our findings on population aging and rural food markets.

## 2 Theoretical analysis and research hypotheses

### 2.1 Theoretical foundations

#### 2.1.1 Agricultural sector

It is assumed that the rural economy primarily relies on agricultural production, operates as a closed system, and is dominated by agricultural activities. The production function follows the classical Cobb-Douglas form, yielding:


$$\begin{array}{l} Y=AK^{1-\beta }L^{\beta } \end{array}$$ (1)

In Equation (1), *Y* represents total agricultural output, A denotes total factor productivity, and *L* signifies effective labor input. Thus, the production function can be rewritten as: *K* represents land and capital. In the short run, we assume *K* is fixed, i.e., $K=\bar{K}$. βdenotes the output elasticity of labor. In the agricultural economic system, L primarily represents the number of young rural laborers *N*_*y*_ , with each young person contributing one unit of labor. Thus, the production function can be reformulated as follows:


$$\begin{array}{l} Y=A{\bar{K}}^{1-\beta }N_{y}^{\beta } \end{array}$$ (2)

#### 2.1.2 Household

Assuming perfect competition in the labor market, where wages W equals the marginal product of labor, we obtain:


$$\begin{array}{l} w=\frac{\partial Y}{\partial L}=\frac{\partial Y}{\partial N_{y}}=\beta A{\bar{K}}^{1-\beta }N_{y}^{\beta -1} \end{array}$$ (3)

The total income *I* of rural households derives from the labor income of young members. Assuming each household has one representative young worker, then:


$$\begin{array}{l} I=wL=\beta A{\bar{K}}^{1-\beta }N_{y}^{\beta -1} \end{array}$$ (4)

Since β−1 < 0, we have:


$$\begin{array}{l} \frac{\partial I}{\partial N_{y}}=\beta \left(\beta -1\right)A{\bar{K}}^{1-\beta }N_{y}^{\beta -2}≺0 \end{array}$$ (5)

From Equation (5), we derive the first proposition:

Proposition 1 (P1): Population aging reduces the number of young people in rural areas, thereby decreasing agricultural labor supply and household income.

This proposition aligns with existing literature (33, 34). Population aging manifests as an increase in the elderly population, whose share of the total population grows progressively larger. Let *N*_*o*_denote the number of elderly individuals, with the total population *N* = *N*_*y*_+*N*_*o*_. Aging implies a decrease in *N*_*y*_, leading to a reduction in L. Crucially, each rural household must also support the elderly. The old-age dependency ratio is *r* = *N*_0_/*N*_*y*_. Aging implies an increase in *r*. From the perspective of actual per capita disposable household income, aging imposes a dependency burden effect (35, 36). We obtain:


$$\begin{array}{l} I=\frac{I_{total}}{1+r}=\frac{I_{total}}{1+N_{o}/N_{y}} \end{array}$$ (6)

#### 2.1.3 Micro-level consumer demand and dietary quality

Suppose rural residents consume two types of food: high-quality food *H*, such as meat, fruits, and dairy products; and low-quality food *L*, such as staple foods. Their prices are *p*_*H*_ and *p*_*L*_, respectively, with *p*_*H*_>*p*_*L*_. Dietary quality Q is defined as the proportion of high-quality food consumed. The following equation can be derived:


$$\begin{array}{l} Q=\frac{p_{H}H}{p_{H}H+p_{L}L} \end{array}$$ (7)

Household preferences are described by the Stone-Geary utility function, which captures both income effects and subsistence consumption levels (37). We can obtain:


$$\begin{array}{l} U\left(H,L\right)=\alpha \ln\;\left(H-\gamma_{H}\right)+\left(1-\alpha \right)\ln\;\left(L-\gamma_{L}\right) \end{array}$$ (8)

In Equation (8), γ_*H*_ and γ_*L*_ denote the subsistence consumption levels for high–quality and low–quality foods, respectively. α(0 < α < 1) is the preference parameter, representing the relative strength of an individual's preference for high-quality food after satisfying subsistence needs. The budget constraint line is:


$$\begin{array}{l} p_{H}H+p_{L}L=I \end{array}$$ (9)

#### 2.1.4 Solving the model under equilibrium conditions

To solve the utility maximization problem, we establish the Lagrange function:


$$\begin{array}{l} \Gamma =\alpha \ln\;\left(H-\gamma_{H}\right)+\left(1-\alpha \right)\ln\;\left(L-\gamma_{L}\right)+\lambda \left(I-p_{H}H-p_{L}L\right) \end{array}$$ (10)

The first-order conditions (FOC) are as follows:


$$\begin{array}{l} \frac{\partial \Gamma }{\partial H}=\frac{\alpha }{H-\gamma_{H}}-\lambda p_{H}=0 \end{array}$$ (11)

$$\begin{array}{l} \frac{\partial \Gamma }{\partial L}=\frac{1-\alpha }{L-\gamma_{L}}-\lambda p_{L}=0 \end{array}$$ (12)

In Equations (11) and (12), the expressions for λ are respectively:


$$\begin{array}{l} \lambda =\frac{\alpha }{p_{H}\left(H-\gamma_{H}\right)} \end{array}$$ (13)

$$\begin{array}{l} \lambda =\frac{1-\alpha }{p_{L}\left(L-\gamma_{L}\right)} \end{array}$$ (14)

Therefore, the model's identity equation is obtained as:


$$\begin{array}{l} \frac{\partial \Gamma }{\partial \lambda }\text{=}I-p_{H}H-p_{L}L=0 \end{array}$$ (15)

Equation (16) can be derived from Equation (15):


$$\begin{array}{l} \frac{\alpha }{p_{H}\left(H-\gamma_{H}\right)}=\frac{1-\alpha }{p_{L}\left(L-\gamma_{L}\right)} \end{array}$$ (16)

Rearranging Equation (16) yields Equation (17):


$$\begin{array}{l} \alpha p_{L}\left(L-\gamma_{L}\right)=\left(1-\alpha \right)p_{H}\left(H-\gamma_{H}\right) \end{array}$$ (17)

Substituting Equation (17) into the budget constraint yields:


$$\begin{array}{l} \alpha \left(I-p_{H}H-p_{L}\gamma_{L}\right)=\left(1-\alpha \right)p_{H}\left(H-\gamma_{H}\right) \end{array}$$ (18)

$$\begin{array}{l} \alpha I-\alpha p_{H}H-\alpha p_{L}\gamma_{L}=\left(1-\alpha \right)p_{H}H-\left(1-\alpha \right)p_{H}\gamma_{H} \end{array}$$ (19)

Moving the term containing H to one side of the equation gives:


$$\begin{array}{l} \alpha I-\alpha p_{L}\gamma_{L}+\left(1-\alpha \right)p_{H}\lambda_{H}=\alpha p_{H}H+\left(1-\alpha \right)p_{H}H=p_{H}H \end{array}$$ (20)

Thus, the demand function for high-quality meals (H) is:


$$\begin{array}{l} H^{*}=\gamma_{H}+\frac{\alpha }{p_{H}}\left(I-S\right) \end{array}$$ (21)

In Equation (21), *S* = *p*_*H*_γ_*H*_+*p*_*L*_γ_*L*_ represents total subsistence expenditure. Similarly, the demand function for low-quality meals is:


$$\begin{array}{l} L^{*}=\gamma_{L}+\frac{1-\alpha }{p_{L}}\left(I-S\right) \end{array}$$ (22)

Substituting both meal qualities into Equation (7) yields:


$$\begin{array}{l} Q=\frac{p_{H}\left[\gamma_{H}+\frac{\alpha }{p_{H}}\left(I-S\right)\right]}{p_{H}\left[\gamma_{H}+\frac{\alpha }{p_{H}}\left(I-S\right)\right]+p_{L}\left[\gamma_{L}+\frac{1-\alpha }{p_{L}}\left(1-S\right)\right]} \end{array}$$ (23)

Simplifying both numerator and denominator yields Equations (24) and (25):


$$\begin{array}{l} p_{H}\gamma_{H}+\alpha \left(I-S\right) \end{array}$$ (24)

$$\begin{array}{l} p_{H}\gamma_{H}+\alpha \left(I-S\right)+p_{L}\gamma_{L}+\left(1-\alpha \right)\left(I-S\right)=\left(p_{H}\gamma_{H}+p_{L}\gamma_{L}\right)\\ \;+\left(I-S\right)=S+\left(I-S\right)=I \end{array}$$ (25)

Thus, the concise expression for the meal quality demand function is:


$$\begin{array}{l} Q\left(I\right)=\frac{p_{H}\gamma_{H}+\alpha \left(I-S\right)}{I}=\alpha +\frac{p_{H}\gamma_{H}-\alpha S}{I} \end{array}$$ (26)

Starting from income's impact on meal quality, we calculate the derivative of *Q* with respect to income I: yielding


$$\begin{array}{l} \frac{dQ}{dI}=-\frac{p_{H}\gamma_{H}-\alpha S}{I^{2}} \end{array}$$ (27)

Substituting total subsistence expenditure S into the numerator yields:


$$\begin{array}{l} p_{H}\gamma_{H}-\alpha S=p_{H}\gamma_{H}-\alpha \left(p_{H}\gamma_{H}+p_{L}\gamma_{L}\right)=p_{H}\gamma_{H}\left(1-\alpha \right)\\ \;-\alpha p_{L}\gamma_{L} \end{array}$$ (28)

Assume that high-quality food becomes a luxury good or at least a normal good beyond subsistence levels, rather than an inferior good. This implies that its expenditure share does not decrease but may even increase as income rises. That is:


$$\begin{array}{l} \frac{dQ}{dI}\geq 0 \end{array}$$ (29)

This implies:


$$\begin{array}{l} p_{H}\gamma_{H}\left(1-\alpha \right)-\alpha p_{L}\gamma_{L}\leq 0 \end{array}$$ (30)

$$\begin{array}{l} \frac{p_{H}\gamma_{H}}{p_{L}\gamma_{L}}\leq \frac{\alpha }{1-\alpha } \end{array}$$ (31)

This condition is intuitively reasonable. It requires that people's preference for high-quality diets, denoted asα/1−α, be sufficiently large to cover its relative survival cost, *p*_*H*_γ_*H*_/*p*_*L*_γ_*L*_ . Therefore, we assume this condition holds and is a strict inequality, such that *dQ*/*dI*≥0 . Consequently, we derive the second proposition:

Proposition 2 (P2): Dietary quality, Q, is an increasing function of income, I.

Population aging implies a decline in the proportion of young people, i.e., a decrease in *N*_*y*_. Combining Propositions 1 and 2 yields the overall effect of population aging on dietary quality:


$$\begin{array}{l} \frac{dQ}{dN_{y}}=\frac{dQ}{dI}\times \frac{\partial I}{\partial N_{y}} \end{array}$$ (32)

Since *dQ*/*dI*≻0 and ∂*I*/∂*N*_*y*_≺0, we have:


$$\begin{array}{l} \frac{dQ}{dN_{y}}≺0 \end{array}$$ (33)

In summary, PA primarily impacts the agricultural sector by reducing the effective labor force and increasing household pension burdens. We constructed a theoretical model that aligns with China's rural economic realities. Through rigorous mathematical derivation, the model shows that population aging reduces total household income while increasing expenditure on eldercare, thereby lowering actual per capita income for rural households. The resulting decline in disposable income forces households to shift their consumption preferences toward cheaper, lower-quality diets, leading to a decrease in dietary quality Q.

#### 2.1.5 The role of rural food markets

Generally, constructing and improving food markets near rural households' residences yields three primary benefits:

1. Price effect. By reducing transportation costs and intermediary steps for food procurement, the actual price of high-quality food decreases, i.e., *p*_*H*_ declines. Lower food prices enhance rural residents' real purchasing power, generating an “income compensation effect” (38). This compensation partially offsets the decline in real per capita income caused by aging.
2. Convenience effect. Improved accessibility to food markets significantly reduces both explicit and implicit costs for rural residents—particularly mobility-impaired elderly—to obtain high-quality food (30). In the model, this manifests as a decrease in subsistence consumption γ_*H*_. The convenience effect (γ_*H*_↓) reduces the fixed expenditure allocated to subsistence consumption in the demand function $H^{*}=\gamma_{H}+\frac{\alpha }{p_{H}\left(I-S\right)}$ , while increasing the relative weight of variable expenditures (39). This amplifies household consumption's responsiveness to price and income changes, enabling the consumption structure $\frac{H^{*}}{{\text{L}}^{*}}$ to shift more rapidly toward high-quality diets (H) when income I faces shocks. Crucially, even with stable or slightly declining income, falling prices alone can directly trigger significant increases in high-quality food consumption.
3. Social networks and information spillover effects. Food markets serve not only as trading venues but also as hubs for information exchange and social networking. Elderly individuals can access market information on nutrition and cooking methods, influenced by peers' consumption behaviors, generating a peer effect that alters their preferences, that is, increasing parameter **α**. A higher **α** yields dual benefits: (1) directly elevating the overall level of the dietary quality function Q; (2) increasing $\frac{\text{d}\text{Q}}{\text{d}\text{I}}$ in the numerator.

Incorporating total survival consumption expenditure S into Equation (27) yields:


$$\begin{array}{l} \frac{dQ}{dI}=\frac{p_{H}\gamma_{H}-\alpha \left(p_{H}\gamma_{H}+p_{L}\gamma_{L}\right)}{I^{2}}=-\frac{p_{H}\gamma_{H}\left(1-\alpha \right)-\alpha p_{L}\gamma_{L}}{I^{2}} \end{array}$$ (34)

The construction of rural food markets has led to a decrease in the price *p*_*H*_ of high-quality diets and the subsistence consumption level γ_*H*_. Meanwhile, we set *M* = *p*_*H*_γ_*H*_(1−α)−α*p*_*L*_γ_*L*_, and then *dQ*/*dI* = −*M*/*I*^2^. We obtain:


$$\begin{array}{l} \frac{\partial M}{\partial p_{H}}=\gamma_{H}\left(1-\alpha \right)≻0 \end{array}$$ (35)

Therefore, we can obtain:


$$\begin{array}{l} \frac{\partial }{\partial p_{H}}\left(\frac{dQ}{dI}\right)=-\frac{1}{I^{2}}\frac{\partial M}{\partial p_{H}}≺0 \end{array}$$ (36)

From Equation (36), it follows that a decrease in the price of high-quality food leads to an increase in the value of *dQ*/*dI*. For *dQ*/*dI*≻0, an increase in its value implies heightened sensitivity of dietary quality Q to income I. Taking the partial derivative with respect to γ_*H*_ yields:


$$\begin{array}{l} \frac{\partial M}{\partial \gamma_{H}}=p_{H}\left(1-\alpha \right)≻0 \end{array}$$ (37)

Therefore, $\partial /\partial \gamma_{H}\left(dQ/dI\right)=-1/I^{2}\partial N/I^{2}\partial \gamma_{H}≺0$. A decrease in the survival consumption γ_*H*_ leads to an increase in the value of *dQ*/*dI* . Therefore, we can draw the following two conclusions:

1. At higher income levels, a greater *dQ*/*dI* means that income growth leads to a substantial improvement in dietary quality.
2. At lower income levels, a greater *dQ*/*dI* means that the deterioration in dietary quality caused by income decline is mitigated.

The above two conclusions can be validated by the following mathematical model:

Assuming no income sensitivity in rural food markets $\frac{dQ}{dI}{|}_{Before}$ with income sensitivity for the rural food market. $\frac{dQ}{dI}{|}_{After}$, and $\frac{dQ}{dI}{|}_{Before}≻\frac{dQ}{dI}{|}_{After}$. When the negative impact of aging on household income causes Δ*I*≺0, the demand for dietary quality also shifts to 为$\Delta Q=\frac{dQ}{dI}•\Delta I$.Since Δ*I*≺0, and $\frac{dQ}{dI}{|}_{After}$ stronger, we obtain:


$$\begin{array}{l} \Delta Q_{After}=\frac{dQ}{dI}{|}_{After}•\Delta I≻\frac{dQ}{dI}{|}_{Before}•\Delta I=\Delta Q_{Before} \end{array}$$ (38)

RMF increase the derivative of dietary quality Q with respect to income *dQ*/*dI*. This implies that dietary quality improves more rapidly as income rises. More importantly, it means that when household real income declines due to aging, the deterioration in dietary quality is significantly reduced.

Overall, by incorporating rural food markets, the demand function expression for dietary quality under the impact of aging is:


$$\begin{array}{l} Q\left(I,T\right)=\alpha \left(T\right)+\frac{p_{H}\left(T\right)\gamma_{H}\left(T\right)-\alpha \left(T\right)S\left(T\right)}{I} \end{array}$$ (39)

In Equation (39), ∂*p*_*H*_/∂*T*≺0,∂γ_*H*_/∂*T*≺0,∂α/∂*T*≻0. RMF mitigate the marginal effect of aging on dietary quality:


$$\begin{array}{l} \frac{\partial }{\partial T}\left|\frac{dQ}{dr}\right|≺0 \end{array}$$ (40)

Equation (40) confirms that as rural food markets develop, the absolute decline in dietary quality *dQ*/*dr* caused by each additional unit of aging (*r*) diminishes.

In summary, the multiple benefits of rural food markets effectively elevate the position of dietary quality Q and smooth the sensitivity curve of dietary quality to income (40). The final outcome is that when aging imposes a negative shock on household real income, causing income I to decline from *I*_1_ to *I*_2_ , the decrease in dietary quality *Q*_*After*_*I*_2_−*Q*_*After*_*I*_1_ in the presence of rural food markets is smaller than the decrease *Q*_*Before*_*I*_2_−*Q*_*Before*_*I*_1_ in the absence of food markets. Thus, we rigorously demonstrate through mathematical analysis that rural food markets exert a positive moderating effect on the negative impacts of aging.

### 2.2 Research hypotheses

Chapter 2.1 demonstrates from the perspective of family consumption decisions by using mathematical models. However, in the real world, the consumption of rural families is still influenced by multiple factors, which are overlooked in the “ideal assumption” of mathematical models. The theoretical basis derived from the rational person assumption has not fully explained how population aging affects family food choice behavior either. This section will once again analyze the impact of aging and the food market on dietary quality by combining mathematical models and individual consumption decisions, in order to obtain the research hypotheses of this paper.

#### 2.2.1 The impact of aging on dietary quality

With the continuous increase in the proportion of the elderly population, aging has a significant impact on the dietary quality of rural households. This negative impact is mainly manifested in the changes of the quality of life and health capital of the elderly group themselves (17), as well as the direct influence on the food resource allocation pattern and nutritional status of the entire family.

The most direct impact of aging on dietary quality is that the decline in household income leads to changes in resource allocation and consumption behavior. According to the life cycle hypothesis, as rural residents enter old age, their wage income from physical labor drops significantly, and the sustainability of family income is challenged, ultimately leading to a decline in long-term income levels (19). The income of the elderly group relies on transfer payments including pension funds and child support. This part of the income flow is low and uncertain, which leads to liquidity constraints and restrictions on household consumption. Insufficient budget constraints have led rural families to tend to prioritize essential goods in their consumption. Meanwhile, as healthcare spending rises rigidly with age, it creates a budget squeeze effect on food spending. Rural elderly families show extremely high price sensitivity in food consumption, and their food consumption structure tends to downgrade. In other words, the elderly will reduce their consumption of high-value, high-nutrient-density animal-based foods such as fish, meat, eggs, milk and fresh fruits and vegetables, and instead increase their consumption of low-cost, high-energy staple foods. This will lead to a decline in dietary diversity and an imbalance in nutritional structure.

In rural areas, due to limited food access channels and the obstruction of obtaining dietary nutrition information, the rising proportion of elderly members often forms a vicious cycle with the decline in the dietary quality of rural households. From the perspective of food sources, the quality of the traditional diet of rural residents largely depends on the food diversity provided by the courtyard economy of self-grown vegetables and poultry. The decline in physical functions among the elderly leads to a reduction in labor supply, significantly weakening the self-sufficiency of non-market-oriented food and cutting off the stable supply channels of low-cost and high-quality food. In terms of food environment perception, the decline in physical functions among the elderly leads to a contraction in their activity radius, significantly reducing their spatial perception of food supply adequacy and making it difficult for them to effectively obtain diverse food resources. Although *PA* does not directly change the physical distance to markets, but it reduces market accessibility through declining physical mobility, increased transport dependence, and a contraction in activity radius. As a result, elderly individuals perceive lower spatial accessibility to diverse food sources, which in turn reduces dietary quality (41). The decline in market accessibility and the reduction in economic income form a double squeeze, weakening their subjective understanding of the spatial accessibility and economic affordability of food, and forcing consumption choices to tilt toward foods with low nutritional value. The large-scale migration of the labor force from rural to urban areas has led to a large number of rural empty-nest elders and changed the cooking behaviors of rural families. The fact that children go out to work leads to the elderly living alone for a long time. The economies of scale of family cooking disappear, and the average cost of preparing diverse meals for one person is high. Psychological factors such as loneliness reduce the intrinsic utility of their pursuit of dietary quality, making it easy for them to develop a consumption behavior of “just making do”, which leads to a further decline in dietary quality. From the perspective of obtaining dietary nutrition information, the insufficient adaptability of the elderly to digital media leads to a narrowing of information acquisition channels, while traditional information dissemination methods are inefficient and difficult to meet the updated demands for modern dietary nutrition knowledge. The decline of cognitive ability and the weakening of learning motivation form a double barrier, intensifying the difficulty of obtaining and applying dietary nutrition information, having a negative impact on the food cognition and consumption decisions of rural residents, and thereby reducing the overall dietary quality of families (42). We put forward the first research hypothesis:

Hypothesis 1 (H1): Population aging has a negative impact on the dietary quality of rural residents.

The theoretical discussion suggests that population aging affects dietary quality through multiple mediating pathways. However, these pathways operate at different levels: economic burden is a household level financial constraint, market accessibility is an individual level mobility constraint, and food supply perception is a broader system level perception. Moreover, aging does not directly change physical market distance but rather reduces accessibility through declining mobility. To capture these distinct mechanisms clearly and avoid conflating different levels of analysis, we disaggregate the mediating role of food environment perception into three parallel sub hypotheses. Each sub hypothesis corresponds to one of the three indicators measured in our empirical analysis: economic affordability, spatial accessibility, and supply sufficiency. We put forward the following three research hypotheses:

Hypothesis 2a (H2a): PA increases the economic burden on rural households through rising healthcare expenditures and reduced labor income, thereby lowering perceived economic affordability of nutrient rich foods and reducing dietary quality.

Hypothesis 2b (H2b): PA reduces market accessibility for elderly individuals due to declining physical mobility and transport dependence, thereby weakening perceived spatial accessibility to diverse food sources and reducing dietary quality.

Hypothesis 2c (H2c): PA reduces the perceived sufficiency of food supply because elderly households, especially those living alone, have limited capacity to seek out diverse food options, thereby lowering dietary quality.

#### 2.2.2 The regulatory role of rural food markets

In new institutional economics, transaction costs refer to all the expenses required to conclude a transaction, including information costs, negotiation costs, contracting costs, supervision costs, and execution costs (43–45). The layout of rural commercial facilities is scattered. Due to their limited mobility, the transportation cost, time cost and physical cost for the elderly to go to the market for shopping are significantly higher than those for the young. The inconvenience of goods has increased the actual transaction cost of obtaining diverse foods, prompting them to rationally choose to make low-frequency and low-diversity purchases in nearby small stores with a single category, thus creating a predicament of “having demand but no ability”. Under the background of urban-rural integration construction, the rural food market is expected to become an important driving force to buffer the negative effects of aging by strengthening food environmental perception and obtaining dietary nutrition information (38). In the market layout planning, efforts have been made to bring market facilities closer to residential areas or set up convenient vegetable stores and chain supermarket outlets at the village level, effectively reducing the physical distance for the elderly. The rural food market has provided significant assistance in alleviating the negative impact of population aging on the dietary quality of rural residents by optimizing the food supply ecosystem between urban and rural areas and innovating the dissemination model of dietary nutrition information.

From the perspective of food environment perception: (1) The development of rural food markets, by driving the upgrading of supporting infrastructure and shaping the food supply chain between urban and rural areas, enables rural residents to access a more diverse range of food types, thereby significantly enhancing the awareness of food supply adequacy. (2) The development of rural food markets has expanded the food circulation network in rural areas, especially the coverage density of various food trading markets and food sales stores has increased, enabling the surrounding residents to integrate into the regional agricultural food system. It not only enhances the spatial accessibility of the food market, but also helps to save food search and transaction costs, thereby positively influencing residents' affordability of high-nutritionally valuable categories. From the perspective of dietary nutrition information acquisition, rural food markets provide convenience for high-quality diet and nutrition improvement. A well-planned market with a steady flow of customers will attract more merchants to settle in, resulting in price and quality competition among merchants. Competitive pressures will push merchants to offer fresher and more diverse products, and may even lead to niche services for older people, such as processed semi-finished products and soft foods. Older people have access to better and richer food options at the same transaction costs. In addition, with the development and prosperity of food markets in towns and townships, various nutrition education activities and publicity channels have gradually increased, providing convenient conditions for promoting the dissemination of dietary nutrition knowledge. The market agglomeration effect that accompanies the diversification of market entities promotes the dissemination of dietary nutrition information, enhances the nutrition awareness and health literacy of local residents, and provides knowledge supply for improving the collection of family food choices and optimizing the family dietary structure. From the perspective of transaction costs, the construction of rural food markets is not merely about building floors and sheds, but rather a fundamental investment aimed at reducing institutional transaction barriers and restoring market functions. It alleviates the negative impacts brought about by aging by reshaping the physical and institutional environment for food access in rural areas and enhancing market accessibility. Based on the mathematical model and the analysis of this part, this paper proposes the following three research hypotheses:

Hypothesis 3 (H3): RFM have a positive impact on the DQRR.

Hypothesis 4 (H4): RFM positively moderates the negative impact of PA on the DQRR.

Hypothesis 5 (H5): RFM affects the DQRR by influencing the acquisition of dietary nutrition information.

### 2.3 Theoretical contribution to food policy and economics

In summary, the theoretical contribution of this study to the Food Policy and Economics literature is threefold. (1) We extend the standard household consumption model by explicitly incorporating population aging as a negative income shock and rural food markets as a buffering mechanism. (2) We identify three economic channels through which food markets operate: reducing the effective price of high quality food, lowering subsistence consumption thresholds, and increasing preference parameters through information spillovers. (3) We demonstrate that the moderating effect of food markets is theoretically predicted to reduce the absolute decline in dietary quality caused by aging. These theoretical insights are directly relevant to policy interventions aimed at improving rural nutrition through market based instruments.

## 3 Research design

### 3.1 Variable settings

#### 3.1.1 Dietary quality

This study referred to the existing literature (7, 46), and used entropy index (EI) and China healthy diet index (CHDI) to measure the dietary quality of rural residents. Entropy index not only considers the richness of food types consumed by a family, but also reflects the consumption balance of various food types through weight distribution, thus providing a comprehensive assessment perspective of family dietary diversity. The Chinese Healthy Diet Index was designed based on the guidelines of the Chinese Dietary Guidelines for Chinese Residents, and assessed dietary quality by quantifying the fit between food group intake and recommended standards (47, 48). As can be seen in Table 1, the results showed that the mean values of EI and CHDI were 1.258 and 3.102, respectively.

**Table 1:** Descriptive statistics of variables.

Variable	Variable definition	Mean	Standard error
QDRR	EI	1.258	0.301
	CHDI	3.102	0.289
PA	Proportion of family members aged 65 and above	0.265	0.391
RFM	Number of food markets within a one-kilometer radius of the township center	39.667	33.629
Sufficiency of supply	Respondents' subjective evaluations of the sufficiency of food supply in the market (0–10, with 10 points indicating the best evaluation)	9.271	1.050
Spatial accessibility	Respondents' subjective evaluations of the spatial accessibility of food in the market (0–10)	9.202	1.190
Economic affordability	The subjective evaluations of the respondents on the economic affordability of food in the market	8.204	1.615
Acquisition of dietary nutrition information	Respondents who actively learn about or collect dietary nutrition-related information = 1, otherwise = 0	0.555	0.497
Gender	The dietary decision-maker is female = 1, otherwise = 0	0.763	0.425
Age	Age of the dietary decision-makers at the time of the interview	56.464	11.045
Education	Dietary decision-makers who have received junior high school education or above = 1, otherwise = 0	0.571	0.495
Nutrition	The deviation value of body mass index for dietary decision-makers	1.288	2.049
Religious belief	For dietary decision-makers, having a religious belief = 1, otherwise = 0	0.019	0.138
Family size	Number of family members	3.163	1.481
Clan network	Number of households of the same clan in the village where the family is located	27.456	49.891
Proportion of children	Proportion of members aged 12 and under in the total family population	0.061	0.124
Proportion of employed workers	Proportion of members working outside the home to the total number of family members	0.282	0.296
Land transfer	If a family has economic behavior of leasing (purchasing) or renting out (selling) land, it equals 1; otherwise, it equals 0	0.517	0.500
Courtyard planting	Diversity of family courtyard planting	3.497	3.090
Livestock and poultry breeding	If a family raises livestock and poultry, it equals 1; otherwise, it equals 0	0.569	0.496
Price shock	If a family is hit by the increase in food prices = 1, otherwise = 0	0.116	0.320
Household income	Annual per capita household income (CNY)	9.615	1.493
Village economic structure	Proportion of per capita non-agricultural income to net income in a village	0.604	0.212
Village population size	Total registered population of the village	1.533	1.066
Village land area	Jurisdictional area of the village	450.866	390.566

#### 3.1.2 Population aging

Drawing on the practices of previous studies (O ‘Neill and Harris, 2025; (49, 50)), this paper adopts the proportion of family members aged 65 and above as the main indicator to measure PA. Compared with the proportion of family members aged 60 and above, this indicator is more in line with the current reality of the extended average life expectancy of Chinese residents and can accurately reflect the impact of age-related factors on dietary quality. As shown in Table 1, the average proportion of members aged 65 and above in the sample families is 26.5%.

#### 3.1.3 Rural food market

Referring to previous studies (16, 30), based on geographic information data, this paper represents the food market in towns and townships by the number of food markets within a one-kilometer radius of the town center. Compared with the accessibility of specific markets, it can better reflect the overall supply capacity of the food market in the region. The choice of a one kilometer radius is justified on two grounds. This radius captures the walkable access zone around the town center, which is the typical range within which elderly residents without motorized transport can conveniently shop for fresh food.

#### 3.1.4 Mechanism variables

This article examines the influence mechanism of population aging and rural food markets on the dietary quality of rural residents from two perspectives: food environment perception and dietary nutrition information acquisition. Based on previous research foundations (51, 52), this paper incorporates three indicators of supply sufficiency, spatial accessibility, and economic affordability in food environmental perception. These indicators are mainly measured by the respondents' subjective evaluations of the sufficiency of food supply, spatial accessibility and economic affordability in the market. They are used to analyze how market-based food environment perception shapes the connection between population aging, rural food markets and the dietary quality of rural residents. The scoring range for the three indicators is set between 1 and 10. The higher the score, the stronger the corresponding subjective cognition. In addition, this paper measures the acquisition of dietary nutrition information by using whether the respondents actively learn about or collect dietary nutrition-related information, effectively distinguishing the progressive relationship between the subjective initiative of information acquisition and the transformation of actual consumption behavior.

#### 3.1.5 Control variables

To reduce the bias problem caused by omitted variables, this study selected a series of control variables (CV) in accordance with the practical methods of related studies (53, 54). This paper controls other variables that may affect the dietary quality of rural residents, including three levels: dietary decision-makers, families, and villages, in the empirical model. (1) We have controlled for individual characteristics related to dietary decision-makers. Such as gender, age, educational level, nutrition and health, and religious belief. (2) This study took into account the control variables at the family level. They mainly include family size, clan network, proportion of children, proportion of employed workers, land transfer, courtyard planting, livestock and poultry breeding, price shock and family income. (3) This study also incorporated village-level characteristic variables into the empirical model, including village economic structure, village population size and village land area.

### 3.2 Empirical model

To examine the impact of PA and RFM on the DQRR, according to the research idea of the published literature (55, 56), we have constructed the following econometric model:


$$\begin{array}{l} DQRR_{i}=a_{0}+a_{1}PA_{i}+a_{2}CV_{i}+\nu_{c}+\varepsilon_{i} \end{array}$$ (41)

In Equation (41), *a*_0_is a constant term, *PA* is population aging, and *QDRR* is the dietary quality of rural residents. *CV* is the information set of control variables, including the characteristics of dietary decision-makers, family characteristics and village characteristics. *v*_*c*_ is the rural fixed effect, ε_*i*_ is the random perturbation term, *a*_1_ and *a*_2_ are the regression coefficients to be estimated, and *i* is the respondent.


$$\begin{array}{l} DQRR_{i}=b_{0}+b_{1}RFM_{i}+b_{1}CV_{i}+\lambda_{c}+\varepsilon_{i} \end{array}$$ (42)

In Equation (42), *b*_0_ is a constant term, *b*_1_ and *b*_2_ are the regression coefficients to be calculated, and λ_*c*_ is the village fixed effect. To verify the regulatory effect of RMF on the negative impact of PA on DQRR, this study constructed an interaction term of two variables.


$$\begin{array}{l} DQRR_{i}=d_{0}+d_{1}PA_{i}+d_{2}RFM_{i}+d_{3}PA_{i}\times RFM_{i}+d_{4}CV_{i}\\ \;+\lambda_{c}+\varepsilon_{i} \end{array}$$ (43)

In Equation (43), *d*_0_ is a constant term, *CV* is the information set of the control variable, λ_*c*_ is the village fixed effect, ε_*i*_ is the random disturbance term, *i* is the respondent, and *d*_1_, *d*_2_, and *d*_3_ are the regression coefficients to be calculated. In this equation, we mainly focus on whether the regression coefficient *d*_3_ of the interaction term PA × RFM meets expectations and whether its significance passes the statistical test. If its regression coefficient is significantly positive, it means that *RFM* is playing a positive moderating role.

To identify the intermediate mechanism by which *PA* and *RFM* affect *QDRR*, we constructed the second-stage regression equations of *PA* and *RFM* respectively according to the two-stage mediating effect program. The two-stage mediating effect model alleviates the endogeneity problem of the traditional three-stage method to a certain extent (57). Equations (41) and (42) are the first-stage regression equations of *PA* and *RFM* respectively.


$$\begin{array}{l} MV_{i}=c_{0}+c_{1}PA_{i}+c_{2}CV_{i}+\lambda_{c}+\varepsilon_{i} \end{array}$$ (44)

$$\begin{array}{l} MV_{i}=e_{0}+e_{1}RFM_{i}+e_{2}CV_{i}+\lambda_{c}+\varepsilon_{i} \end{array}$$ (45)

In Equations (44) and (45), *MV* is a mediating variable, and *c*_0_ and *e*_0_ are constant terms. In the second-stage equation, we focus on regressing whether the regression coefficients of *PA* and *RFM* with respect to the mediating variables are in line with expectations and whether their significance has passed the statistical test. In Equation (44), *MV* mainly consists of supply sufficiency, spatial accessibility and economic affordability. In Equation (45), MV mainly involves the acquisition of dietary nutrition information. Considering the possible parameter estimation bias caused by heteroscedasticity in cross-sectional data, robust standard errors are adopted throughout the regression analysis process in this paper.

### 3.3 Data sources

The data used in this article is derived from a micro-survey of rural residents' food consumption conducted by the author in Shandong, Jiangxi and Gansu provinces from May to August 2024. The sample selection takes into account both the balance of geographical distribution and the differences in rural income gradients. We adopt a stratified random sampling framework. We determined the sampling frame through county-level cluster analysis and successively selected 12 counties (districts), 36 towns and 108 administrative villages, ultimately obtaining 1,080 valid samples of rural households. To ensure the reliability of the sample data, this survey adopted a face-to-face interview format, covering both the village level and the household level. Village data is mainly collected through interviews with village cadres, including information such as village population, economic structure, and infrastructure construction, covering a total of 108 villages. The data content of farmers covers variables such as family population structure and educational level to ensure a relatively comprehensive portrayal of the basic characteristics of farmers. In terms of obtaining food consumption data, this study adopted the method of recording food consumption for three consecutive days. The classification standards for food types refer to the “Chinese Dietary Guidelines for Residents,” covering multiple categories such as grains, tubers, and legumes, which can comprehensively reflect the dietary quality of rural residents. In addition, the data of the food market in towns and townships is derived from the collection of local geographic information data and on-site research of sample town and township markets, obtaining the food market data of 36 towns and townships. Descriptive statistical analysis of each variable is shown in Table 1.

## 4 Results

### 4.1 Benchmark regression results

We first estimate the baseline effects of population aging and rural food markets on dietary quality. This step addresses the first part of our core research question. The regression results of *PA* and the impact of *RFM* on the *DQRR* are shown in Table 2. The F-values of each column are all significant at the 1% statistical level, indicating that the model constructed in this paper has a good explanatory ability. As shown in Table 2, under the condition of keeping other variables unchanged, *PA* has a significant negative impact on the *DQRR*. Its regression coefficients were −0.101 and −0.172 respectively, and it passed the statistical significance test. H1 has been verified. The results in columns (2) and (4) show that the regression coefficients of *RFM* are 0.007 and 0.05 respectively, and both are significant at the 1% level. This result implies that the food market will significantly improve the *DQRR*. H3 has been verified. The results in columns (3) and (6) show that the interaction coefficient between *PA* and the *RFM* is 0.001 and 0.002 respectively, both of which are significant at the 5 and 1% levels respectively. This result implies that *RFM* can, to a certain extent, alleviate the negative impact of *PA* on the *DQRR*. H4 was verified.

**Table 2:** The result of the benchmark regression.

Variable	EI			CHDI		
	(1)	(2)	(3)	(4)	(5)	(6)
PA	−0.101^***^		−0.092^**^	−0.172^***^		−0.152^***^
	(−3.52)		(−2.09)	(−4.02)		(−3.46)
RFM		0.007^**^	0.004^*^		0.005^**^	0.003^*^
		(2.42)	(2.03)		(1.99)	(1.74)
*PA*×*DFM*			0.001^**^			0.002^***^
			(2.36)			(5.91)
Gender	0.022	0.017	0.015	0.065^**^	0.051^**^	0.043^**^
	(0.99)	(0.82)	(0.75)	(2.24)	(2.21)	(2.42)
Age	−0.006	−0.003	−0.001	0.001	0.002	0.000
	(−1.48)	(−1.35)	(−1.27)	(1.02)	(1.00)	(0.99)
Education	0.088^***^	0.075^***^	0.067^***^	0.046^*^	0.042^*^	0.036^*^
	(3.91)	(3.52)	(3.19)	(1.90)	(1.75)	(1.88)
Nutrition	−0.006	−0.005	−0.005	−0.009	−0.008	−0.007
	(−1.09)	(−1.16)	(−1.12)	(−1.40)	(−1.43)	(−1.34)
Religious belief	−0.086^*^	−0.084^*^	−0.083^*^	0.054	0.058	0.040
	(−1.73)	(−1.72)	(–.69)	(0.91)	(0.95)	(0.78)
Family size	0.024^***^	0.021^***^	0.019^**^	0.021^**^	0.025^**^	0.016^**^
	(2.99)	(3.20)	(2.72)	(2.52)	(2.06)	(2.29)
Clan network	0.009	0.002	0.000	0.003	0.002	0.000
	(1.04)	(1.07)	(1.02)	(0.95)	(0.58)	(0.83)
Proportion of children	0.198^**^	0.166^**^	0.171^**^	0.056	0.052	0.045
	(2.58)	(2.45)	(2.32)	(0.82)	(0.60)	(0.79)
Proportion of employed workers	−0.351	−0.033	−0.027	0.021	0.016	0.009
	(−1.07)	(−0.90)	(−0.92)	(0.62)	(0.62)	(0.42)
Land transfer	0.016	0.014	0.013	0.026	0.035	0.022
	(1.26)	(0.81)	(0.79)	(1.44)	(1.49)	(1.30)
Courtyard planting	0.017^*^	0.012	0.004	0.008^*^	0.006^*^	0.005^*^
	(1.76)	(1.43)	(1.22)	(1.82)	(1.77)	(1.73)
Livestock and poultry breeding	0.097^***^	0.092^***^	0.077^***^	0.082^***^	0.089^***^	0.060^***^
	(4.95)	(3.50)	(3.85)	(3.91)	(4.52)	(3.57)
Price shock	0.008	0.009	0.004	0.042	0.057	0.026
	(0.67)	(0.69)	(0.18)	(1.11)	(1.25)	(0.80)
Household income	0.022^**^	0.035^**^	0.016^**^	0.005	0.003	0.003
	(2.22)	(2.61)	(1.98)	(0.55)	(0.50)	(0.37)
Village economic structure	−0.055	−0.045	−0.043	−0.082	−0.069	−0.052
	(−1.53)	(−1.42)	(−1.47)	(−0.67)	(−0.75)	(−0.49)
Village population size	0.101^*^	0.081	0.089	0.092	0.092	0.089
	(1.68)	(1.47)	(1.54)	(1.52)	(1.48)	(1.42)
Village land area	−0.003	−0.002	−0.000	−0.005	−0.001	−0.000
	(1.25)	(−0.99)	(−1.01)	(−1.40)	(−1.03)	(−1.11)
Village fixed effect	Yes	Yes	Yes	Yes	Yes	Yes
Constant term	0.896^**^	0.952^***^	0.871^**^	3.652^***^	2.902^***^	2.873^***^
	(2.46)	(3.94)	(2.45)	(6.95)	(4,52)	(3.79)
F-statistic	5.804^***^	6.985^***^	5.605^***^	4.562^***^	4.021^***^	3.694^***^
R-square	0.319	0.314	0.327	0.216	0.259	0.280
Observed value	1,080	1,080	1,080	1,080	1,080	1,080

^***^, ^**^ and ^*^ respectively indicate significance at the 1, 5 and 10% levels, and the t-statistic is reported in parentheses.

In addition, control variables such as the educational level of dietary decision-makers, family size, and livestock and poultry breeding will also have a certain impact on the *DQRR*. Firstly, the educational level of dietary decision-makers has a significant positive impact on the *DQRR*, indicating that rural families with higher educational levels of dietary decision-makers have higher dietary quality levels. The possible reason lies in that dietary decision-makers with higher educational levels tend to purchase diversified and nutrient-rich foods to optimize the family dietary structure. Secondly, family size has a significant promoting effect on the *DQRR*. This may stem from the fact that families with more members have more economies of scale advantages in food acquisition and consumption, and can choose more diverse foods in the market at a lower unit food cost (12). Finally, the impact of livestock and poultry breeding on the *DQRR* is also significantly positive, indicating that conducting livestock and poultry breeding activities in families is conducive to improving the dietary quality of rural residents' families. The possible reason is that the animal protein food produced by livestock and poultry breeding activities provides a stable material basis for improving dietary quality (14).

### 4.2 Mechanism analysis

To uncover why population aging reduces dietary quality and why rural food markets improve it, we test two mediating pathways. The first pathway is food environment perception, including supply sufficiency, spatial accessibility, and economic affordability. The second pathway is the acquisition of dietary nutrition information. The empirical analysis results in the previous text indicate that the food market in towns and townships helps alleviate the negative impact of population aging on the dietary quality of rural residents. Existing studies have shown that food environmental perception, including supply adequacy, spatial accessibility, and economic affordability, is closely related to residents' food choices and dietary quality (59). This section further explores the potential mechanisms by which aging and the food market influence *DQRR* from the perspectives of food environment perception and dietary nutrition information acquisition.

The results in columns (1) to (3) of Table 3 show that *PA* has a negative impact on rural residents' perceptions of food supply adequacy, spatial accessibility, and economic affordability. These results support H2a, H2b, and H2c respectively. Specifically, population aging reduces perceived economic affordability (H2a), spatial accessibility (H2b), and supply sufficiency (H2c), each of which contributes to lower dietary quality. The results in column (4) show that the regression coefficient of *RFM* on the acquisition of dietary impact information is 0.017 and is significant at the 10% level. This result implies that building food markets in towns and townships will enhance rural families' access to more dietary imaging information. H5 has been tested.

**Table 3:** The result of the mechanism test.

Variable	Sufficiency of supply	Spatial accessibility	Economic affordability	Acquisition of dietary nutrition information
	(1)	(2)	(3)	(4)
PA	−0.508^***^	−0.361^*^	−0.307^***^	
	(−2.58)	(1.87)	(−4.92)	
RFM				0.017^*^
				(1.82)
*CV*	Yes	Yes	Yes	Yes
Village fixed effect	Yes	Yes	Yes	Yes
Observed value	1,080	1,080	1,080	1,080

^***^ and ^*^ respectively indicate significance at the 1 and 10% levels, and the t-statistic is reported in parentheses.

A valid question: does the budget squeeze from rising healthcare costs truly outweigh the decline in caloric needs of the elderly? We argue that it does, for three reasons. (1) While basal metabolic rate and total caloric requirements decline with age, the demand for nutrient density, including protein, vitamins, and minerals, does not decline and may even increase due to physiological absorption inefficiencies and chronic disease management. Therefore, the relevant margin is not calorie sufficiency but dietary quality. A budget squeeze that reduces consumption of expensive, nutrient rich foods (meat, eggs, dairy, fresh produce) while maintaining or increasing staple food intake leads to a decline in dietary quality even if total calories remain adequate (58). (2) Healthcare costs in rural China exhibit a rigid upward trend with age, particularly for chronic conditions such as hypertension and diabetes. These expenditures are largely non discretionary. In contrast, food expenditure is more flexible. When healthcare costs rise, households rationally adjust by compressing food budgets, especially the share allocated to high quality foods. This substitution effect is well documented in the health economics literature on household resource allocation. (3) Our empirical results in Table 3 show that population aging significantly reduces perceived economic affordability (coefficient = −0.307, p < 0.01). This subjective measure captures the real pressure that elderly households face when allocating limited income between healthcare and nutrition. Taken together, these arguments support the interpretation that economic affordability is a genuine mediating pathway, not a spurious correlation driven by declining caloric needs.

### 4.3 Heterogeneity analysis

To identify for whom the effects are strongest, we examine heterogeneity across three social structure variables: gender of the dietary decision maker, strength of family clan networks, and village population size. This analysis informs targeted food policy design. In the benchmark regression section, the variables we used to divide the samples were incorporated into the empirical model. To reduce the interference caused by collinearity, the control variables used for sample division in the heterogeneity analysis section are excluded from the regression equation. For instance, in the section of gender heterogeneity testing, we excluded the gender of dietary decision-makers, while other control variables were included in the model. All subsequent heterogeneity analyses adopted this method and utilized cluster-robust standard errors.

#### 4.3.1 Gender heterogeneity of dietary decision-makers

It can be seen from Table 4 that in the sample where the dietary decision-makers are male, the regression coefficients of *PA* are −0.105 and −0.173, and it passes the 1% significance test. The regression coefficients of *RFM* were 0.008 and 0.009, and they were statistically significant at the 1 and 5% levels, respectively. In the sample where the dietary decision-makers were female, the regression coefficients of *PA* were −0.099 and −0.154, and were significant at the 5 and 1% levels, respectively. The regression coefficients of *RFM* are 0.004 and 0.005, and they are significant at the 10 and 5% levels respectively. From these data, it can be found that compared with families where the dietary decision-makers are women, the influence of *PA* and *RFM* on *DQRR* is more intense in the male sample. From the perspective of the interaction terms, the regression coefficients of the male samples were 0.006 and 0.021 respectively, and both passed the significance test at the 1% level. The regression coefficients in the female sample were 0.002 and 0.007, and both were significant at the 5% level. By comparing their coefficients, the regression coefficient of PA × RFM in the male sample is significantly greater than that in the female sample. This result indicates that in families where men dominate dietary decisions, the buffering effect of *RFM* on the negative effects of *PA* is more significant.

**Table 4:** The results of the gender heterogeneity test.

Variable	EI		CHDI	
	Male	Female	Male	Female
	(1)	(2)	(3)	(4)
*PA*	−0.105^***^	−0.099^**^	−0.173^***^	−0.154^***^
	(−3.93)	(−2.22)	(−4.57)	(−4.02)
*RFM*	0.008^***^	0.004^*^	0.009^**^	0.005^**^
	(2.99)	(1.78)	(2.51)	(2.19)
*PA*×*RFM*	0.006^***^	0.002^**^	0.021^***^	0.007^**^
	(5.20)	(2.32)	(7.47)	(2.16)
*CV*	Yes	Yes	Yes	Yes
Village fixed effect	Yes	Yes	Yes	Yes
Observed value	653	427	653	427

^***^, ^**^ and ^*^ respectively indicate significance at the 10, 5 and 1% levels. The t-statistic is reported in parentheses.

The reasons for these results may be as follows: (1) Differentiated dietary preferences. Traditional social norms have shaped different gender roles in diet. Men are usually regarded as the main bearers of heavy physical labor, and they need meat such as meat and high-fat foods to replenish their energy. Women are more closely associated with light foods such as vegetables and grains. Therefore, the negative impact of PA on men is stronger. As men age and their physical strength declines, their ability to obtain high-energy and high-protein foods from self-produced foods decreases, which has a more direct and significant impact on their traditional eating habits. In families where decision-making is dominated by women or for women themselves, they may have long been accustomed to making adjustments under limited resources, and their dietary patterns themselves are more resilient and sustainable. In the face of the impact of PA, they may first respond by adjusting their food structure and household expenditure rather than immediately turning to the market for purchases. (2) Decision-making authority and resource allocation. In many traditional rural families, men are usually the main source of the family's cash income. This enables men to have stronger purchasing power and willingness to pay when facing the food market. When aging leads to a decline in the ability to produce food independently, male decision-makers can more directly and effectively utilize the market to replace and supplement household food supplies, and purchase more diverse and higher-nutrition commercial foods. (3) Transaction costs and market access. Men tend to participate in public domain activities more frequently, including visiting town markets. They have lower transaction costs for obtaining market information, negotiating prices, and long-distance transportation. In contrast, female decision-makers may be constrained by mobility, social norms or the burden of family care, resulting in higher market access costs and thus weakening their ability to use the market to cushion the negative impacts of an aging population. These differences in gendered behavioral patterns enable the supply potential of rural food markets to be more fully transformed into actual improvements in dietary quality in male-decision-making families.

#### 4.3.2 Heterogeneity of clan networks

This study divided the sample into strong and weak samples based on the average number of households of the same clan in the village where the family was located to analyze the heterogeneity generated by the clan network. It can be seen from Table 5 that in the samples of strong clan networks, the regression coefficients of QDRR of *PA* are −0.115 and −0.196 respectively, and both have passed the 1% significance test. In the samples of weak clan networks, the regression coefficients of *PA* were −0.186 and −0.252 respectively, and both were significant at the 1% level. This result implies that the dietary quality of families in weak clan network samples is more susceptible to the negative impact of aging. Judging from the results of *RFM*, its positive effect on QDRR is more pronounced in families with stronger clan networks. Its regression coefficient is greater than that of the weak clan network. From the perspective of the moderating effect of *RFM*, the regression coefficients in the strong family network samples are 0.019 and 0.016, and both are significant at the 5% level. The regression coefficients of the weak clan network are 0.027 and 0.036, and they are significant at the 5 and 1% levels respectively. This result implies that the mitigating effect of *RFM* on the impact of aging is more prominent in families with weak clan networks. The reasons for these results may lie in the following aspects.

1. Mutual assistance network and risk-sharing mechanism. In rural China, family-based elderly care and clan mutual assistance are traditional and important forms of informal elderly care security. In villages with weak clan networks, this mutual assistance system based on blood ties and geographical ties is underdeveloped. When families are confronted with problems such as a reduction in the labor force, health issues and a decline in income caused by an aging population, they are unable to rely on clan networks to receive food gifts, labor assistance or economic support. Meanwhile, the formal social security system in rural China, such as pensions and long-term care services, is still not well-developed. Therefore, they are exposed to the risk of a lack of dual safeguards, and the quality of their diet is naturally more vulnerable to impact. A strong clan network is like a risk pool. When a family encounters difficulties, risks can be shared within the clan. Furthermore, strong clan networks are often associated with better local social capital, which may indirectly help members obtain better economic opportunities and thereby increase their willingness to pay for high-quality meals.
2. Information advantage and trust mechanism. The information flow within a strong clan network is fast, and based on blood ties and long-term interactions, the level of trust is high. Market information such as where to buy high-quality and low-priced food and which vendors have a good reputation can be disseminated more efficiently within the clan. This information spillover effect and trust transfer reduce the uncertainty and cost of market transactions, enabling them to make better use of the market.
3. Resource allocation in clan networks. In strong clan network families, the food exchange and gift-giving mechanism within the clan, especially the food support system for elderly families, weakens the buffering effect of the rural food market on the decline in dietary quality caused by population aging. For families with weak clan networks, due to the lack of traditional social security networks, the market is their main choice to cope with the impact of an aging population. Rural food markets, by offering a diverse supply of ingredients, can only be highly relied upon and utilized when the problem of an aging population arises. Therefore, once the market experiences a “timely help” like a buffer effect, it becomes extremely prominent and significant.

**Table 5:** The results of the heterogeneity test of the clan network.

Variable	EI		CHDI	
	Strong	Weak	Strong	Weak
	**(1)**	**(2)**	**(3)**	**(4)**
*PA*	−0.115^***^	−0.186^***^	−0.196^***^	−0.252^***^
	(−3.51)	(−4.95)	(−3.62)	(−5.85)
*RFM*	0.021^*^	0.035^**^	0.016^**^	0.022^***^
	(2.10)	(2.53)	(1.99)	(3.82)
*PA*×*RFM*	0.019^**^	0.027^**^	0.014^**^	0.036^***^
	(2.38)	(2.16)	(2.45)	(6.28)
*CV*	Yes	Yes	Yes	Yes
Village fixed effect	Yes	Yes	Yes	Yes
Observed value	448	632	448	632

^***^, ^**^ and ^*^ respectively indicate significance at the 10, 5 and 1% levels. The t-statistic is reported in parentheses

#### 4.3.3 Heterogeneity in population size

This study, based on the average population size of the villages where the respondents are located, divides the sample into two samples: large households and small households, to analyze the heterogeneity brought about by population size. The results of the population size heterogeneity test are shown in Table 6.

**Table 6:** The results of the heterogeneity test for village size.

Variable	EI		CHDI	
	Big	Small	Big	Small
	(1)	(2)	(3)	(4)
PA	−0.056^**^	−0.079^**^	−0.073^*^	−0.093^***^
	(−1.79)	(−2.50)	(−1.72)	(−3.11)
RFM	0.010^**^	0.025^***^	0.013^*^	0.019^**^
	(2.36)	(4.96)	(1.81)	(2.54)
*PA*×*RFM*	0.003^*^	0.011^***^	0.007^**^	0.016^***^
	(1.79)	(3.42)	(2.11)	(3.98)
CV	Yes	Yes	Yes	Yes
Village fixed effect	Yes	Yes	Yes	Yes
Observed value	463	671	463	671

^***^, ^**^ and ^*^ respectively indicate significance at the 10, 5 and 1% levels.

The t-statistic is reported in parentheses.

In the administrative village sample with a larger population size, the regression coefficients of *PA* for *DQRR* were −0.056 and −0.073 respectively, and they were significant at the 5 and 10% levels respectively. In another sample, the regression coefficients of *PA* were −0.079 and −0.093, and they were significant at the 5 and 1% levels respectively. By comparing their regression coefficients, it can be found that *PA* has a greater negative impact on dietary quality in the sample of administrative villages with smaller population sizes. From the regression coefficient of *RFM* to *DQRR*, it can be seen that its positive effect is more intense in the administrative village sample with a smaller population size. For instance, the results of columns (1) and (2) indicate that the regression coefficients of *RFM* are 0.010 and 0.025 respectively, and they are significant at the 5 and 1% levels respectively. The coefficient of the latter is significantly greater than that of the former. From the perspective of the moderating effect of *RFM*, in the sample of villages with a larger population size, the regression coefficients of the interaction term are 0.003 and 0.007, and they are significant at the 10 and 5% levels, respectively. In another sample, its regression coefficients are 0.011 and 0.016, and both were significant at the 1% level. This result implies that the effect of *RFM* in alleviating the negative impact of *PA* is more pronounced in villages with smaller population sizes.

The reasons for these results may lie in the following aspects. (1) Differences in the initial conditions of commercial market facilities. Small villages suffer from more severe market failures and the absence of public services, which makes their internal buffering capacity to cope with the impact of an aging population extremely weak. Large-scale villages usually come with or are close to regional markets and have relatively mature commercial ecosystems. Their commercial competition is relatively intense, food accessibility is high, and residents are accustomed to obtaining diverse foods through the market. However, small-scale villages lack commercial infrastructure. Usually, there are only one or two small stores with a single category of products. Their functions are mainly limited to providing cigarettes, alcohol, snacks and basic daily necessities, and they almost have no capacity to supply fresh food. The quality of residents' diets is highly dependent on self-production and self-sale as well as long-distance procurement. Due to the inherent market fragility of small villages, families in the villages show more obvious vulnerability when facing the impact of aging, making the negative impact more severe. (2) Differences in marginal benefits. The construction of food markets in small villages has generated higher marginal utility and achieved Pareto improvement. Building food markets in small-scale villages directly transforms residents' food acquisition model from high transaction cost external dependence to low transaction cost community availability. The convenience, diversity improvement and transaction cost reduction brought about by this breakthrough development from scratch have made the marginal return rate of the market built in small villages higher. Villages with larger population sizes may have more food alternative networks, such as mobile vendors and periodic markets, thereby diluting the potential of rural food markets to improve the dietary quality of rural residents. (3) The food market directly alleviates the food transaction costs in small villages, thereby more effectively blocking the transmission path of the aging impact. In small villages, the construction of food markets directly reconnects the elderly in the village, who have been locked in by high transaction costs, into the food supply chain. This has enabled the reactivation and compensation of their purchasing power that was deprived by aging. In large-scale villages, market construction can indeed reduce transaction costs. However, since the elderly already have a certain ability to access the market, this reduction is more of an improvement in convenience rather than a change in survival.

### 4.4 Robustness test

#### 4.4.1 Replace the core explanatory variables

(1) Considering the crucial role of dietary decision-makers in shaping family dietary habits and the impact of age on family food consumption decisions, this paper uses whether family dietary decision-makers are 65 years old or above as a surrogate variable for *PA*. (2) To avoid measurement deviations of food markets in towns and townships as much as possible, this paper uses the number of food markets on the main shopping streets of the sample towns in the survey data to re-examine the development of food markets in towns and townships. The results in Table 7 show that after changing the measurement method of the core explanatory variable, *RFM* can still alleviate the negative impact of *PA* on the *DQRR*, verifying the robustness of the benchmark regression results.

**Table 7:** The results of the robustness test.

Variable	EI		CHDI	
	(1)	(2)	(3)	(4)
*PA*	−0.185^***^		−0.242^***^	
	(−4.04)		(−5.42)	
*RFM*		0.009^***^		0.004^**^
		(2.84)		(2.10)
CV	Yes	Yes	Yes	Yes
Village fixed effect	Yes	Yes	Yes	Yes
Observed value	1,080	1,080	1,080	1,080

^***^ and ^**^ respectively indicate significance at the 1% and 5% levels, and the t-statistic is reported in parentheses.

#### 4.4.2 Causal identification

The quality of diet itself may also affect the aging process. For instance, better nutrition may extend life expectancy, thereby exacerbating aging. This reverse causality will lead to deviations in the results. To enhance the accuracy of the estimation results, this paper selects the proportion of the population aged 0–14 in the county where the village is located during the third national census conducted by the Chinese government in 1982 as the instrumental variable (IV) for aging. Population transition is a long-term process. The high birth rate several decades ago would form a large specific birth cohort, which would inevitably enter old age several decades later, directly determining the current aging rate. This is a powerful and mechanical predictive relationship. Therefore, it satisfies the principle of relevance. (2) In China, the population structure after the 1950s was mainly determined by the national policies, social traditions and mortality rates at that time, and had nothing to do with the current short-term economic fluctuations that affect the quality of rural diets, modern health awareness, the development of the food market, etc. Therefore, it satisfies the principle of exogeneity. As shown in Table 8, the regression coefficient of stage IV in the first phase is −0.442 and is significant at the 1% level, indicating that the correlation condition is met. In the relevancy test of the IV, the p-value of the Anderson canonical correlation LM statistic is less than 0.01, rejecting the null hypothesis of insufficient instrumental variable identification. Cragg-Donald Wald F statistic is 78.07, and is greater than the corresponding Stock-Yogo critical value of 16.38, rejecting the null hypothesis of a weak instrumental variable. These three results indicate that the selected IV is appropriate. Judging from the results of columns (2) and (3). The regression coefficients of *PA* were −0.146 and −0.221 respectively, and both were significant at the 1% level. This result confirms the conclusion in Table 2 that PA negatively affects RDQ.

**Table 8:** Result of the endogeneity test.

Variable	PA	DQRR	DQRR	RFM	DQRR	DQRR
	(1)	(2)	(3)	(4)	(5)	(6)
PA		−0.146^***^	−0.221^***^			
		(−8.29)	(−8.95)			
RFM					0.035^***^	0.027^***^
					(3.90)	(3.69)
IV	−0.442^***^			0.371^***^		
	(−3.72)			(3.85)		
CV	Yes	Yes	Yes	Yes	Yes	Yes
Village fixed effect	Yes	Yes	Yes	Yes	Yes	Yes
F-statistic	78.07			106.26		
LM statistic	242.841^***^			350.952^***^		

^***^ and ^**^ respectively indicate significance at the 1% and 5% levels. The t-statistic is reported in parentheses in columns (1) and (4), while the Z-statistic is reported in parentheses in the remaining results.

The development of rural food markets can influence the food consumption decisions of rural residents. The upgrading of local residents' food consumption demands may force the supply capacity of food markets in towns and townships to increase. There may be endogeneity problems caused by reverse causality between the food markets in hometown towns and the dietary quality of rural residents. In addition, due to unobservable factors such as individual dietary preferences and regional food culture, they may simultaneously affect the food market in towns and townships and the dietary quality of rural residents. Therefore, in this paper, the average values of comprehensive supermarkets and rural food markets in other towns of this county are selected as instrumental variables for the rural food market. The reasons are as follows: (1) Within the same county, each township faces similar policy environments, transportation infrastructure and consumption cultures. There is a significant spatial dependence in the location decisions of different market entities, and different types of food markets have internal connections in shaping the local food supply chain. Therefore, it meets the requirements of relevance. (2) Within a county, there are dual barriers of administrative boundaries and market radiation ranges among different towns. Rural residents usually purchase food in their local towns rather than others (16). Therefore, the selected IV only affects the *DQRR* through the local town food market, meeting the exogeneity requirement. As can be seen from the results in column (4) of Table 8, the regression coefficient of the first stage IV is 0.371 and is significant at the 1% level, indicating that the correlation condition has been verified. In the relevancy test of the IV, the p-value of the Anderson canonical correlation LM statistic is less than 0.01, rejecting the null hypothesis of insufficient instrumental variable identification. Cragg-Donald Wald F statistic is 106.26,and is greater than the corresponding Stock-Yogo critical value of 16.38, rejecting the null hypothesis of a weak instrumental variable. These three results indicate that the selected IV is appropriate. According to the results in columns (5) and (6) of Table 8, the regression coefficients of *RFM* in the second stage are 0.035 and 0.027 respectively, and both are significant at the 1% level. This result once again confirms the conclusion in Table 2 that *RFM* will positively affect *DQRR*.

#### 4.4.3 Sensitivity analysis of buffer radius

To address the concern that a one kilometer radius may underestimate market access in rural areas, we re estimated our main models using alternative buffer radii of 3 kilometers and 5 kilometers. The 3 kilometer radius approximates a 30–50 minute walk or a 10 minute bicycle ride, which is feasible for many elderly residents. The 5 kilometer radius represents a short electric bike or bus ride, typical for weekly or biweekly shopping trips in more dispersed villages. These two range radii also conform to the research design of the existing literature (16). Table 9 reports the results. Panel A uses the 3 kilometer radius, and Panel B uses the 5 kilometer radius. In both panels, the coefficients for *RFM* remain positive and statistically significant at the 1 or 5% level. The interaction term *PA* × *RFM* also remains positive and significant. Therefore, our main conclusions are robust to the choice of buffer radius.

**Table 9:** Sensitivity analysis of buffer radius.

Variable	EI		CHDI	
	Panel A (3 km radius)		Panel B (5 km radius)	
	(1)	(2)	(3)	(4)
*PA*	−0.089^**^	−0.148^***^	−0.035^*^	−0.139^***^
	(−2.12)	(−3.21)	(−1.77)	(−3.05)
*RFM*	0.005^**^	0.004^*^	0.003^*^	0.003^*^
	(2.15)	(1.92)	(1.87)	(1.79)
*PA × RFM*	0.0007^**^	0.0009^***^	0.0006^**^	0.0007^**^
	(2.18)	(5.12)	(2.01)	(2.45)
CV	Yes	Yes	Yes	Yes
Village fixed effect	Yes	Yes	Yes	Yes
Observed value	1,080	1,080	1,080	1,080

^***^, ^**^, ^*^ respectively indicate significance at the 1%, 5%, and 1% level, and the t-statistic is reported in parentheses.

The slightly smaller coefficients observed with the 3 km and 5 km buffers are expected. Larger radii include more peripheral markets that are less accessible to elderly individuals with mobility constraints. While these markets exist within a 5 km radius, many elderly residents cannot easily reach them without motorized transport. Therefore, the 1 km buffer better captures the actual food environment that shapes daily dietary choices. The persistence of significant coefficients across all three radii confirms that our main findings are robust, but the declining magnitude highlights that proximity matters. Policy interventions should focus on bringing food markets within walking distance of elderly populations.

## 5 Conclusions and policies

### 5.1 Conclusion

Against the backdrop of an aging rural population, seeking the dynamic mechanism to improve the dietary quality and family welfare of rural residents is of great practical significance for addressing the challenges of an aging society and enhancing the level of healthy human capital in rural areas. This study utilized micro-survey data to empirically examine the mechanism by which population aging and rural food markets affect the dietary quality of rural residents. Our study differs from earlier research in several important ways. Previous studies, such as Sibhatu et al. (10) and Huang and Tian (12), focused on income and production diversity but overlooked the moderating role of market infrastructure. Unlike Zeng et al. (16) who examined only direct effects, we explicitly test how RFM buffers the PA DQRR relationship. Furthermore, while Zheng et al. (13) emphasized nutrition knowledge, we identify three parallel mechanisms of food environment perception that have not been jointly analyzed. The heterogeneity analysis by gender, clan networks, and village size also provides novel insights absent in prior literature. We have reached the following conclusions: (1) *PA* has had a significant negative impact on the *DQRR*, while the rural food market can effectively promote the improvement of the *DQRR*. This result once again confirms the conclusions of the published literature (30, 38). (2) *PA* has weakened rural families' perception of the food environment. These capabilities mainly include supply sufficiency, spatial accessibility and economic affordability. (3) *RFM* have enhanced families' ability to obtain dietary nutrition information, thereby improving the quality of meals. (4) *RFM* have a positive moderating effect on the negative impact of *PA*. This conclusion implies that building more food markets in towns and villages will be beneficial for families to withstand the negative impacts brought about by *PA*. (5) The impact of *PA* and *RFM* on the *DQRR* shows group heterogeneity. The influence of *PA, RFM* and moderating effects is more intense in samples where the family diet decision-makers are male, small-scale villages and weak clan networks.

This study advances the existing discourse within the Food Policy and Economics. While prior research has documented the negative association between aging and nutrition, our study provides a novel theoretical mechanism: the positive moderating role of rural food markets. We show that food market development does not merely increase food access in general terms but specifically buffers the dietary decline associated with aging related income and mobility constraints. This finding shifts the policy conversation from individual level nutrition education toward market based structural interventions. Furthermore, our heterogeneous analysis reveals that the buffering effect is strongest in villages with small populations and weak clan networks, suggesting that food market investments can serve as a compensatory mechanism where traditional social support is lacking. The necessity of this study lies in its policy relevance. As rural China ages rapidly, understanding how market based interventions can compensate for demographic disadvantages is urgent. Our findings demonstrate that strengthening township food markets is not merely a consumption issue but a strategic tool to mitigate aging related nutritional decline, especially in areas with weak social networks or small village populations.

### 5.2 Policy implications

The research conclusions of this paper are conducive to providing policy references and decision-making basis for further balancing the efficiency of market resource allocation with the development goals of social inclusiveness.

(1) Government departments should build an elderly-friendly market system and enhance the supply response capacity. Promote supply-side reform in township markets and plan special sales areas for elderly-friendly food. Provide rent subsidies for elderly-friendly stalls and guide merchants to optimize their product category structure. At the same time, market transformation should be integrated into the construction of the county-level commercial system. Through the optimization of the transportation network, the market accessibility of the elderly group should be enhanced to form a demand oriented supply system. This market based approach can be further complemented by community based self-sufficiency initiatives discussed below.

(2) Establish a nutrition information empowerment network to break through the bottleneck of cognitive constraints. Establish a visual nutrition guidance system in township markets and develop dietary procurement guidelines for chronic diseases. Rely on village-level organizations to carry out intergenerational nutrition knowledge transmission training, with a focus on enhancing the information processing capabilities of female decision-makers. Integrate the digital rural platform to push personalized dietary plans and precisely convey nutritional knowledge and guide behaviors through short videos and other media. In functioning food markets, consumers learn from merchant demonstrations, product displays, and peer shopping behavior. When such markets are absent, short videos can artificially recreate these information spillover effects by showing how to select, prepare, and consume nutrient rich foods. Evidence from digital health interventions in low resource settings supports the feasibility of this approach. Therefore, policy makers should consider investing in village level digital literacy training for elderly residents and supporting local content creation tailored to regional dietary cultures and common chronic diseases.

(3) Implement differentiated institutional interventions to eliminate structural constraints. In areas with weak clan networks, the focus of relief policies should be on vigorously developing and improving the rural food market system. For example, improving logistics, building markets, and ensuring food safety. In regions with strong clan networks, policies can focus more on popularizing nutrition knowledge and guiding healthy consumption, and attempt to cooperate with existing clan organizations to integrate modern nutrition concepts into traditional mutual assistance systems. When implementing nutrition improvement programs or providing food subsidies, it is necessary to recognize the systematic differences in behavioral patterns between male and female decision-makers. For male decision-makers, emphasis can be placed on providing market information and guiding healthy consumption. For female decision-makers, it is necessary to consider how to reduce their market transaction costs and enhance their economic empowerment.

(4) The construction of rural food markets, such as the location of the market, opening hours, and transportation convenience, should fully take into account the needs of women, reduce their burden of participating in the market, and enable this positive factor of the market to benefit all types of families more fairly.

(5) In cases where resources are limited, priority should be given to supporting the construction of food market systems in villages with small populations and remote geographical locations.

(6) In addition to strengthening township food markets, policy makers should also consider complementary interventions that enhance local resource self-sufficiency, particularly for elderly individuals who face severe mobility constraints and cannot easily travel to town centers. Government support for community gardens and shared courtyards can serve as a decentralized, low cost supplement to formal market access. For example, village collectives or local governments could allocate small plots of land for community gardens where elderly residents can grow fresh vegetables collectively, reducing their reliance on distant markets. Similarly, revitalizing idle homestead plots or shared courtyards for small scale planting by neighboring elderly households can provide a stable source of nutrient rich produce. These local production initiatives are especially valuable in remote villages where market construction is economically infeasible in the short term. Moreover, such programs can be integrated with the formal food market system through organized periodic deliveries of market sourced supplements, such as eggs, dairy, and meat, to community garden participants. This multi layered strategy combines market infrastructure with community based self sufficiency, offering a more resilient response to the dietary challenges posed by population aging in rural areas

### 5.3 Limitations

This study has several limitations that point to promising avenues for future research. One notable limitation is that our survey did not collect data on the use of short video platforms or other digital media for nutrition information acquisition. As a result, we cannot empirically test the potential role of short videos in bridging the nutrition information gap, particularly in small villages where traditional food markets are absent. Nevertheless, we believe this mechanism merits theoretical discussion and future empirical investigation. Short videos may serve as a low cost, scalable tool to compensate for missing market based information spillovers through at least three pathways. First, short videos can overcome spatial barriers by delivering visual, easy to understand nutrition guidance directly to elderly residents via smartphones, bypassing the need for physical market presence. Second, in small villages with strong social ties, short videos shared among villagers can generate observational learning and peer effects, amplifying the reach of nutrition messages. Third, where food markets are absent, short videos can artificially recreate the information spillover effects that normally occur through merchant demonstrations, product displays, and peer shopping behavior in functioning markets. We therefore call for future research to design surveys and experiments that explicitly measure exposure to short video content, assess its impact on dietary knowledge and behavior, and examine whether it can buffer the negative dietary consequences of population aging in rural areas. Such research would complement the market based interventions emphasized in this study and provide a more complete policy portfolio for improving the dietary quality of vulnerable aging populations.

Another limitation concerns our interpretation of the economic affordability mechanism. We argue that rising healthcare costs create a budget squeeze that reduces the ability of elderly households to purchase nutrient rich foods, thereby lowering dietary quality. However, it is also true that total caloric requirements decline with age due to reduced basal metabolic rate and physical activity. A natural and important question: does the budget squeeze from healthcare costs truly outweigh the decline in caloric needs? Our current dataset does not contain detailed information on household healthcare expenditure, caloric intake, or specific nutrient consumption. Therefore, we cannot empirically decompose the relative contributions of these two opposing forces. Future research should collect detailed longitudinal data on both healthcare spending and dietary intake at the individual level. Such data would allow researchers to test whether the budget squeeze effect dominates the caloric needs decline in shaping dietary quality among aging populations. Specifically, studies could examine whether the reduction in spending on high quality foods (meat, eggs, dairy, produce) persists even after adjusting for lower caloric requirements. We call for future work to address this important question, as it has direct implications for whether policy interventions should focus on income support, healthcare cost reduction, or nutrition education for the elderly.

## Data Availability

Publicly available datasets were analyzed in this study. This data can be found here: The raw data supporting the conclusions of this article will be made available by the authors, without undue reservation.
